# Association between weight, weight perception, weight teasing and mental health among adolescents

**DOI:** 10.1186/s13034-024-00730-2

**Published:** 2024-03-23

**Authors:** Wenxin Gu, Xiaoyan Yu, Yinliang Tan, Zhiping Yu, Jingfen Zhu

**Affiliations:** 1 School of Public Health, Shanghai Jiao Tong University, Shanghai, China; 2 Department of Social Medicine and Health Education, School of Public Health, Peking University, Beijing, China; 3 Department of Nutrition and Dietetics, University of North Florida, Jacksonville, FL USA

**Keywords:** Body weight, Mental health, Adolescent

## Abstract

**Background:**

Adolescent mental health problems are becoming increasingly prevalent, and there are correlations between weight-related concerns and adolescent mental health. The aim of this study is to explore the association between three weight-related factors (actual weight, weight perception, and weight teasing) and mental health problems (depressive symptoms, anxiety symptoms, and loneliness) in Chinese adolescents.

**Methods:**

10,070 adolescents between the ages of 11–18 from schools in Shanghai, China were selected using a stratified random cluster sampling method. Self-reported questionnaires were collected to investigate weight-related factors and mental health problems. Logistic regression analysis was used to examine the relationship.

**Results:**

The prevalence of depressive symptoms, loneliness, mild anxiety symptoms, and moderate to severe anxiety symptoms among adolescents were 18.0%, 53.8%, 26.5%, and 12.3%, respectively, with a higher prevalence found in females. After adjusting for weight perception and weight teasing, actual weight had no harmful impact on adolescents’ mental health. Adolescents' perception of being overweight increased the risk of depressive symptoms, loneliness, mild anxiety symptoms, and moderate to severe anxiety symptoms, while the perception of being underweight had a similar but more profound impact (depressive symptoms OR = 1.590, 95% CI: 1.342–1.883; loneliness OR = 1.537, 95% CI: 1.353–1.746; mild anxiety symptoms OR = 1.368, 95% CI: 1.178–1.589; moderate to severe anxiety symptoms OR = 1.780, 95% CI: 1.449–2.186). Experiencing weight teasing more than once a year had a greater effect on adolescents' mental health, especially among adolescents with overweight/obesity (depressive symptoms OR = 2.970, 95% CI: 2.325–3.793; loneliness OR = 3.839, 95% CI: 3.119–4.727; mild anxiety symptoms OR = 2.822, 95% CI: 2.236–3.562; moderate to severe anxiety symptoms OR = 5.212, 95% CI: 3.846–7.065).

**Conclusions:**

The prevalence of mental health problems among adolescents was high, especially loneliness. Weight perception and weight teasing, but not the actual weight, independently influenced adolescent mental health.

## Introduction

The 2019 Global Burden of Disease Report indicated that mental disorders ranked within the top 10 global disease burdens, which is a health concern among children and adolescents as well. More than 225 million children and adolescents were estimated to suffer from mental disorders worldwide [1]. Depression and anxiety are two common psychological problems. The prevalence of depressive and anxiety symptoms among adolescents in China was 43.7% and 37.4%, respectively [2]. Mental disorders in adolescents are associated with a variety of adverse consequences, including increased susceptibility to social exclusion, stigmatization, risk-taking behaviors, and deteriorated physical health [3]. Loneliness is a negative emotion that occurs when people are dissatisfied with their social relationships [4]. Loneliness peaks in adolescence and gradually declines in adulthood [5]. An alarming 11%-20% of adolescents aged 12–15 reported feeling lonely at least "sometimes" [6]. There may be a reciprocal effect between loneliness and symptoms of depression and anxiety in children and adolescents [7]. Adolescent mental health problems have become increasingly prominent and require more attention.

Rapid physical development and growth occur during adolescence [8], and these changes in the body may make teenagers more self-conscious about their weight. The association between different weight-related factors (actual weight, weight perception, and weight teasing) and adolescent mental health has been explored separately. According to the World Health Organization, obesity rates have nearly tripled worldwide since 1975 [9]. In 2019, the prevalence of overweight and obesity among Chinese children and adolescents was 11.1% and 7.9%, respectively [10]. Obesity has been identified as a significant independent risk factor for adolescent mental health [11]. Research has shown that obese children and adolescents are more prone to develop symptoms of depression, anxiety, and loneliness [12, 13]. Findings on the relationship between weight perception (the individual's assessment of his/her actual weight status [14]) and adolescent mental health were inconsistent. A large Norwegian study revealed that perceiving oneself as underweight or overweight was significantly associated with symptoms of depression and anxiety in adolescents [15]. Underweight perception was also found to be associated with an increased risk of mental health problems only among boys [16]. In the context of Chinese adolescents, there was a positive correlation between overweight perception and psychological symptoms [17]. Moreover, a notable association was observed between body dissatisfaction and increased reports of loneliness [18]. When people's perceptions of their weight are different from their actual weight status, this is referred to as weight misperception [19]. Children and adolescents with weight misperception had higher risks of psychological distress [20]. Weight teasing, a form of weight stigma, has been demonstrated to be detrimental to children's and adolescents' mental health and approximately 13%-32% of adolescents experienced discrimination because of their weight [21]. Studies have indicated that the experience of discrimination and stigmatization was associated with higher levels of depression, anxiety, and loneliness [22]. Among adolescents, those who experienced weight-based teasing had a higher level of depression [23]. Youth from immigrant communities, similar to the general population, also suffered from adverse emotional health outcomes associated with weight-based teasing [24]. Additionally, a gradient relationship was observed between the frequency of weight-related teasing and the occurrence of psychosomatic symptoms in school-aged youth [25].

In a cross-sectional study of 57,059 Canadian adolescents, the association between three weight-related factors and mental health were examined together and the results showed that deviating from normal weight perception and experiencing bullying independently predicted symptoms of depression and anxiety, while actual weight did not. These findings were true regardless of gender [14]. However, there is still a lack of research on the effects of actual weight, weight perception, and weight teasing on adolescent mental health problems (depressive symptoms, anxiety symptoms, and loneliness). Moreover, the majority of studies have concentrated on adolescents' depression and/or anxiety, with the influence of weight-related factors on loneliness relatively under-researched. This study examined the association between three weight-related factors (actual weight, weight perception, and weight teasing) and mental health problems (depressive symptoms, anxiety symptoms, and loneliness) among adolescents in Shanghai, China. The objective was to gain a deeper insight into the risk factors for adolescent mental health and to identify potential effective interventions to improve the mental well-being of adolescents.

## Methods

### Study design and population

This cross-sectional study was carried out in public schools which participated in the 2021 longitudinal cohort of the Youth Health Behavior Survey in Shanghai, China. A representative sample of high school students were selected by a stratified random cluster sampling method in May 2021. 3 out of 16 districts in Shanghai were selected randomly, and 21 schools (12 junior middle schools, 6 senior middle schools, and 3 vocational middle schools) in the 3 districts were selected at random according to school types. All of the 10,070 students in the chosen schools were invited to complete the questionnaire. Ethics approval was granted by the Ethics Committee of the School of Public Health, Shanghai Jiao Tong University (SJUPN-202016). Written informed consents from all the participants (and their guardians) were obtained.

### Data collection

Students were invited to complete an anonymous online questionnaire in class groups. The procedure was carried out by a qualified investigator, who gave participants instructions on how to fill out the questions after first outlining the goal. Participants received assistance and clarification from qualified investigators as well. 9194 valid questionnaires were included in the final analysis after 876 surveys from adolescents were removed due to missing weight or height data and inadequate completion time. The response rate was 91.30%.

### Measurements

#### Actual weight

Actual weight was measured by Body Mass Index (BMI), which was calculated from self-reported height (cm) and weight (kg). Participants were divided into "Underweight," "Normal," and "Overweight/obese" groups according to the screening standards for malnutrition, overweight, and obesity in Chinese school-aged children and adolescents [26, 27].

#### Weight perception

Weight perception was assessed by asking "How would you describe your weight?" The responses were divided into three groups: "underweight", "about the right weight" and "overweight".

#### Weight teasing

Weight teasing was assessed by asking "Have you ever been teased about your weight?". The responses were "never", "less than once a year", "several times a year", "several times a month", and "at least once a week". Given that experiencing weight teasing is not the same as never experiencing it, and experiencing weight teasing a few times a year or more was associated with negative physical and psychological outcomes [28], responses were categorized into three groups: "never", "less than once a year" and "more than once a year". Among these, "more than once a year" was defined as "several times a year," "many times a month," and "at least once a week."

#### Depressive symptoms

The Chinese Version of the Patient Health Questionnaire-2 (PHQ-2-C) was used to measure depressive symptoms which has a good reliability index for screening depressive disorders in Chinese adolescents, with a Cronbach's α coefficient of 0.849. The rating scale ranges from 0 to 3, the sum of the scores of the two items is the total score, and a total score of 3 or more indicates depressive disorder [29].

#### Anxiety symptoms

The Generalized Anxiety Disorder-7 (GAD-7) was adopted to assess anxiety symptoms. The Cronbach's α coefficient was 0.956. There are seven elements in total, with values ranging from 0 to 3, denoting none, a few days, more than half, and almost every day, respectively. A final score is calculated by summing the contributions (0–21 points). < 5, 5–9, 10–14, 15–21 [30], respectively represent the degree of no, mild, moderate, and severe anxiety symptoms. This study categorized anxiety into three categories for analysis: no, mild, and moderate to severe due to the low prevalence of severe anxiety symptoms among adolescents.

#### Loneliness

The UCLA Loneliness Scale Short Version (ULS-6) was used to assess loneliness. Studies have demonstrated the validity of the ULS-6 in Chinese adolescents, with a Cronbach's α coefficient of 0.955. The scale has six entries with a range of 1 to 4, which correlate to never, seldom, sometimes, and always. Higher total scores indicate higher levels of loneliness [31]. Those with a total score of ≤ 6 were defined as having no loneliness and ≥ 7 as having loneliness.

#### Covariates

Covariates included sex (male or female), school type (junior middle school, senior middle school, and vocational middle school), monthly pocket money (< 200, 200–599, and ≥ 600 CNY), whether living on campus, and academic performance (top 25%, middle, and bottom 25%).

### Statistical analysis

IBM SPSS 26.0 statistical software was used for data analysis. Descriptive statistics including numbers and percentages were used. Chi-squared tests were performed to compare socio-demographic characteristics among sex and different mental health problems. Logistic regression was used to analyze the effects of different weight-related factors on mental health. Because the test of parallel lines of anxiety symptoms did not meet the requirement of ordinal logistic regression (*P* < 0.05), multinomial logistic regression was used to investigate the effects of weight-related factors on anxiety symptoms, with the no anxiety symptoms group as the reference. For depressive symptoms and loneliness, binary logistic regression was used, with the groups without depressive symptoms and loneliness serving as the reference. In model 1, the sociodemographic characteristics (sex, school type, living situation, academic performance, and pocket money) were adjusted. Besides that, BMI (actual weight), weight perception and weight teasing were added in model 2. To explore the potential differences among sex and BMI, sex and BMI stratification analyses were conducted based on Model 2. *P* < 0.05 was considered statistically significant for the differences in the tests performed.

## Results

### Participant characteristics

Table 1 shows the participants’ demographic characteristics. Of the 9,194 adolescents, 55.3% were boys and 44.7% were girls. Nearly half of the participants were junior high school students, constituting 47.5% of the total. 63.0% of individuals have a normal BMI, with 9.2% underweight and 27.8% classified as overweight/obese. Girls had a lower percentage of overweight/obese compared to boys (18.2% vs 35.6%). Boys were more likely to perceive themselves as underweight than girls (24.5% vs 13.3%), while girls tended to have more overweight perception than boys (47.1% vs 40.5%). 30.5% of adolescents experienced weight teasing, with a higher prevalence among girls than boys (less than once a year: 15.1% vs 12.8%; more than once a year: 18.0% vs 15.6%).

**Table 1:** Characteristics of the sample, n (%)

		Total	Male	Female	*χ*^2^	*P*
School type	Junior middle school	4367 (47.5)	2287 (45.0)	2080 (50.6)	302.718	< 0.001
	Senior middle school	2682 (29.2)	1268 (25.0)	1414 (34.4)		
	Vocational middle school	2145 (23.3)	1526 (30.0)	619 (15.0)		
Live at school	Yes	1496 (16.3)	882 (17.4)	614 (14.9)	9.856	0.002
	No	7698 (83.7)	4199 (82.6)	3499 (85.1)		
Academic performance	Top quarter	3267 (35.5)	1864 (36.7)	1403 (34.1)	23.586	< 0.001
	Middle	4274 (46.5)	2249 (44.2)	2025 (49.2)		
	Bottom quarter	1653 (18.0)	968 (19.1)	685 (16.7)		
Pocket money	< 200 CNY	4804 (52.2)	2684 (52.8)	2120 (51.5)	24.731	< 0.001
	200–599 CNY	2930 (31.9)	1525 (30.0)	1405 (34.2)		
	≥ 600 CNY	1460 (15.9)	872 (17.2)	588 (14.3)		
BMI	Underweight	841 (9.2)	519 (10.2)	322 (7.8)	402.351	< 0.001
	Normal	5795 (63.0)	2753 (54.2)	3042 (74.0)		
	Overweight/obese	2558 (27.8)	1809 (35.6)	749 (18.2)		
Weight perception	Underweight	1792 (19.5)	1244 (24.5)	548 (13.3)	180.888	< 0.001
	About the right weight	3407 (37.0)	1781 (35.0)	1626 (39.6)		
	Overweight	3995 (43.5)	2056 (40.5)	1939 (47.1)		
Weight teasing	Never	6393 (69.5)	3640 (71.6)	2753 (66.9)	23.924	< 0.001
	Less than once a year	1270 (13.8)	648 (12.8)	622 (15.1)		
	More than once a year	1531 (16.7)	793 (15.6)	738 (18.0)		
Total		9194 (100)	5081 (55.3)	4113 (44.7)		

### Distributions of depressive symptoms, loneliness, and anxiety symptoms among adolescents with different sociodemographic characteristics

Table 2 shows that the prevalence of depressive symptoms, loneliness, mild anxiety symptoms, and moderate to severe anxiety symptoms among adolescents in Shanghai, China was 18.0%, 53.8%, 26.5%, and 12.3%, respectively, with girls being more likely than boys to have mental health problems. Senior middle school students had the highest rates of depressive symptoms, loneliness, and anxiety symptoms, followed by junior middle school students, while vocational middle school students had the lowest rates. Students living at school were more likely to experience loneliness (56.90%), while the prevalence of depressive symptoms was higher among students not living at school (18.30%). The prevalence of depressive symptoms was highest (19.70%) among adolescents who were overweight/obese. In terms of weight perception, adolescents who perceived themselves as overweight were more likely to experience mental health problems than those who perceived themselves as underweight (depressive symptoms: 21.80% vs. 18.80%; loneliness: 60.30% vs. 54.40%; mild anxiety symptoms: 30.30% vs. 26.20%; moderate to severe anxiety symptoms: 15.20% vs. 12.90%). The prevalence of mental health problems among adolescents increased as the number of experiences of weight teasing increased. The prevalence of depressive symptoms, loneliness, mild anxiety symptoms, and moderate to severe anxiety symptoms among adolescents who experienced weight teasing more than once a year reached 32.70%, 73.00%, 34.00%, and 25.30%, respectively.

**Table 2:** Distributions of depressive symptoms, loneliness, and anxiety symptoms among adolescents with different sociodemographic characteristics, n (%)

		Depressive symptoms			Loneliness			Anxiety symptoms				
		yes	*χ*^2^	*P*	yes	*χ*^2^	*P*	no	mild	moderate to severe	*χ*^2^	*P*
Sex	Male	817 (16.1)	27.793	< 0.001	2450 (48.2)	140.457	< 0.001	3279 (64.5)	1259 (24.8)	543 (10.7)	58.933	< 0.001
	Female	836 (20.3)			2493 (60.6)			2343 (57.0)	1181 (28.7)	589 (14.3)		
School type	Junior middle school	795 (18.2)	66.990	< 0.001	2179 (49.9)	188.094	< 0.001	2958 (67.7)	913 (20.9)	496 (11.4)	337.682	< 0.001
	Senior middle school	585 (21.8)			1738 (64.8)			1259 (46.9)	994 (37.1)	429 (16.0)		
	Vocational middle school	273 (12.7)			1026 (47.8)			1405 (65.5)	533 (24.8)	207 (9.7)		
Live at school	Yes	241 (16.1)	4.235	0.040	851 (56.9)	7.004	0.008	875 (58.5)	445 (29.7)	176 (11.8)	9.426	0.009
	No	1412 (18.3)			4092 (53.2)			4747 (61.7)	1995 (25.9)	956 (12.4)		
Academic performance	Top quarter	475 (14.5)	103.938	< 0.001	1669 (51.1)	38.365	< 0.001	2096 (64.2)	826 (25.3)	345 (10.6)	60.335	< 0.001
	Middle	744 (17.4)			2277 (53.3)			2639 (61.7)	1119 (26.2)	516 (12.1)		
	Bottom quarter	434 (26.3)			997 (60.3)			887 (53.7)	495 (29.9)	271 (16.4)		
Pocket money	< 200 CNY	879 (18.3)	17.119	< 0.001	2542 (52.9)	3.787	0.151	3033 (63.1)	1198 (24.9)	573 (11.9)	25.652	< 0.001
	200–599 CNY	468 (16.0)			1617 (55.2)			1754 (59.9)	832 (28.4)	344 (11.7)		
	≥ 600 CNY	306 (21.0)			784 (53.7)			835 (57.2)	410 (28.1)	215 (14.7)		
BMI	Underweight	145 (17.2)	6.826	0.033	433 (51.5)	1.984	0.371	525 (62.4)	213 (25.3)	103 (12.2)	5.570	0.234
	Normal	1005 (17.3)			3124 (53.9)			3550 (61.3)	1561 (26.9)	684 (11.8)		
	Overweight/obese	503 (19.7)			1386 (54.2)			1547 (60.5)	666 (26.0)	345 (13.5)		
Weight perception	Underweight	337 (18.8)	95.121	< 0.001	975 (54.4)	155.626	< 0.001	1092 (60.9)	469 (26.2)	231 (12.9)	175.373	< 0.001
	About the right weight	446 (13.1)			1560 (45.8)			2354 (69.1)	761 (22.3)	292 (8.6)		
	Overweight	870 (21.8)			2408 (60.3)			2176 (54.5)	1210 (30.3)	609 (15.2)		
Weight teasing	Never	904 (14.1)	291.654	< 0.001	2967 (46.4)	464.337	< 0.001	4333 (67.8)	1481 (23.2)	579 (9.1)	521.124	< 0.001
	Less than once a year	248 (19.5)			859 (67.6)			666 (52.4)	438 (34.5)	166 (13.1)		
	More than once a year	501 (32.7)			1117 (73.0)			623 (40.7)	521 (34.0)	387 (25.3)		
Total		1653 (18.0)			4943 (53.8)			5622 (61.1)	2440 (26.5)	1132 (12.3)		

### Associations between actual weight, weight perception, weight teasing and adolescents’ mental health

As shown in Table 3, in Model 1, being overweight/obese was positively associated with depressive symptoms (OR = 1.218, 95%CI: 1.077–1.379,* P* = 0.002), loneliness (OR = 1.134, 95% CI: 1.029–1.251, *P* = 0.011), and moderate to severe anxiety symptoms (OR = 1.268, 95% CI: 1.093–1.470, *P* = 0.002) among adolescents. However, in Model 2, actual weight had no harmful impact on mental health problems and overweight/obese even showed a protective effect against loneliness, mild anxiety symptoms, and moderate to severe anxiety symptoms. In Model 2, compared to adolescents who perceived themselves as about the right weight, those having the perception of being overweight were more vulnerable to experiencing depressive symptoms (OR = 1.311, 95% CI: 1.132–1.518, *P* < 0.001), loneliness (OR = 1.260, 95% CI: 1.128–1.407, *P* < 0.001), mild anxiety symptoms (OR = 1.282, 95% CI: 1.129–1.456, *P* < 0.001), and moderate to severe anxiety symptoms (OR = 1.364, 95% CI: 1.141–1.630, *P* = 0.001), while underweight perception also increased the risk of various mental health problems and had a greater impact than overweight perception (depressive symptoms OR = 1.590, 95% CI: 1.342–1.883, *P* < 0.001; loneliness OR = 1.537, 95% CI: 1.353–1.746, *P* < 0.001; mild anxiety symptoms OR = 1.368, 95% CI: 1.178–1.589, *P* < 0.001; moderate to severe anxiety symptoms OR = 1.780, 95% CI: 1.449–2.186,* P* < 0.001). Adolescents who had experienced weight teasing were more likely to have mental health problems compared to those who had not. Moreover, the effects of experiencing weight teasing more than once a year on adolescents' depressive symptoms (OR = 2.759, 95% CI: 2.388–3.189, *P* < 0.001), loneliness (OR = 3.138, 95% CI: 2.740–3.593, *P* < 0.001), mild anxiety symptoms (OR = 2.522, 95% CI: 2.181–2.917, *P* < 0.001), and moderate to severe anxiety symptoms (OR = 4.671, 95% CI: 3.922–5.563, *P* < 0.001) were greater compared to adolescents who experienced weight teasing less than once a year. Overall compared to actual weight, weight perception and weight teasing had a stronger association with depressive symptoms, loneliness, and anxiety symptoms in adolescents.

**Table 3:** Associations between actual weight, weight perception, weight teasing and adolescents' mental health

		**Depressive symptoms**		**Loneliness**		**Mild Anxiety symptoms**		**Moderate to Severe Anxiety symptoms**	
		OR(95% CI)	*P*	OR(95% CI)	*P*	OR(95% CI)	*P*	OR(95% CI)	*P*
**Model 1**									
BMI	Normal	ref		ref		ref		ref	
	Underweight	1.033 (0.850–1.255)	0.743	0.982 (0.847–1.139)	0.814	0.958 (0.805–1.139)	0.625	1.078 (0.856–1.357)	0.523
	Overweight/obese	1.218 (1.077–1.379)	0.002	1.134 (1.029–1.251)	0.011	1.044 (0.932–1.169)	0.456	1.268 (1.093–1.470)	0.002
Weight perception	About the right weight	ref		ref		ref		ref	
	Underweight	1.596 (1.363–1.869)	< 0.001	1.533 (1.363–1.725)	< 0.001	1.376 (1.198–1.582)	< 0.001	1.813 (1.499–2.194)	< 0.001
	Overweight	1.764 (1.554–2.004)	< 0.001	1.701 (1.547–1.870)	< 0.001	1.569 (1.405–1.753)	< 0.001	2.085 (1.788–2.432)	< 0.001
Weight teasing	Never	ref		ref		ref		ref	
	Less than once a year	1.419 (1.212–1.661)	< 0.001	2.302 (2.022–2.621)	< 0.001	1.815 (1.582–2.082)	< 0.001	1.761 (1.452–2.136)	< 0.001
	More than once a year	2.824 (2.480–3.216)	< 0.001	3.073 (2.712–3.482)	< 0.001	2.488 (2.177–2.842)	< 0.001	4.600 (3.934–5.379)	< 0.001
**Model 2**									
BMI	Normal	ref		ref		ref		ref	
	Underweight	0.905 (0.731–1.121)	0.362	0.863 (0.733–1.015)	0.076	0.895 (0.740–1.083)	0.253	0.912 (0.706–1.177)	0.477
	Overweight/obese	0.888 (0.772–1.021)	0.096	0.796 (0.711–0.892)	< 0.001	0.758 (0.666–0.863)	< 0.001	0.794 (0.670–0.942)	0.008
Weight perception	About the right weight	ref		ref		ref		ref	
	Underweight	1.590 (1.342–1.883)	< 0.001	1.537 (1.353–1.746)	< 0.001	1.368 (1.178–1.589)	< 0.001	1.780 (1.449–2.186)	< 0.001
	Overweight	1.311 (1.132–1.518)	< 0.001	1.260 (1.128–1.407)	< 0.001	1.282 (1.129–1.456)	< 0.001	1.364 (1.141–1.630)	0.001
Weight teasing	Never	ref		ref		ref		ref	
	Less than once a year	1.388 (1.177–1.636)	< 0.001	2.323 (2.030–2.658)	< 0.001	1.815 (1.573–2.095)	< 0.001	1.768 (1.446–2.163)	< 0.001
	More than once a year	2.759 (2.388–3.189)	< 0.001	3.138 (2.740–3.593)	< 0.001	2.522 (2.181–2.917)	< 0.001	4.671 (3.922–5.563)	< 0.001

Model 1 was adjusted by sex, school type, accommodation status, academic performance, and pocket money

Model 2 included sex, school type, accommodation status, academic performance, pocket money, BMI (actual weight), weight perception and weight teasing

### Associations between actual weight, weight perception, weight teasing and adolescents' mental health by sex

As shown in Table 4, after implementing sex stratification, both underweight (OR = 1.731, 95% CI: 1.323–2.265, *P* < 0.001) and overweight perception (OR = 1.464, 95% CI: 1.202–1.784, *P* < 0.001) of girls increased the risk of depressive symptoms compared with those who perceived themselves as about the right weight. In boys, the risk of depressive symptoms was increased only when they perceived themselves as underweight (OR = 1.461, 95% CI: 1.172–1.821, *P* < 0.001). Compared to girls who had never experienced weight teasing, those who experienced weight teasing less than once a year (OR = 1.543, 95% CI: 1.229–1.937,* P* < 0.001) and more than once a year (OR = 2.745, 95% CI: 2.231–3.378, *P* < 0.001) were more likely to have depressive symptoms. Meanwhile, only boys who experienced weight teasing more than once a year had an increased risk of depressive symptoms (OR = 2.801 95% CI: 2.285–3.434, *P* < 0.001), while those who experienced weight teasing less than once a year did not (*P* > 0.05).

**Table 4:** Associations between actual weight, weight perception, weight teasing and adolescents' mental health by sex, OR (95% CI)

		Depressive symptoms		Loneliness		Mild Anxiety symptoms		Moderate to Severe Anxiety symptoms	
		Male	Female	Male	Female	Male	Female	Male	Female
BMI	Normal	ref	ref	ref	ref	ref	ref	ref	ref
	Underweight	0.871 (0.656–1.156)	0.994 (0.715–1.381)	0.789^*^ (0.639–0.973)	1.025 (0.787–1.333)	1.007 (0.790–1.284)	0.758 (0.555–1.034)	0.729 (0.512–1.039)	1.215 (0.834–1.768)
	Overweight/obese	0.957 (0.784–1.168)	0.841 (0.683–1.036)	0.760 ^***^ (0.654–0.884)	0.856 (0.711–1.030)	0.760^**^ (0.637–0.906)	0.734^**^ (0.598–0.900)	0.776^*^ (0.608–0.990)	0.788 (0.613–1.014)
Weight perception	About the right weight	ref	ref	ref	ref	ref	ref	ref	ref
	Underweight	1.461^***^ (1.172–1.821)	1.731 ^***^ (1.323–2.265)	1.496 ^***^ (1.274–1.756)	1.558 ^***^ (1.255–1.935)	1.264 ^*^ (1.044–1.532)	1.573^***^ (1.229–2.013)	1.698^***^ (1.297–2.223)	1.898 ^***^ (1.371–2.626)
	Overweight	1.132 (0.905–1.416)	1.464 ^***^ (1.202–1.784)	1.235 ^*^ (1.052–1.449)	1.313^**^ (1.124–1.533)	1.306^**^ (1.081–1.577)	1.263^**^ (1.061–1.504)	1.351^*^ (1.028–1.777)	1.381^**^ (1.087–1.755)
Weight teasing	Never	ref	ref	ref	ref	ref	ref	ref	ref
	Less than once a year	1.248 (0.980–1.590)	1.543 ^***^ (1.229–1.937)	2.342 ^***^ (1.955–2.807)	2.337^***^ (1.907–2.864)	1.792^***^ (1.469–2.185)	1.874^***^ (1.522–2.309)	1.570 ^**^ (1.169–2.107)	2.047^***^ (1.545–2.712)
	More than once a year	2.801^***^ (2.285–3.434)	2.745^***^ (2.231–3.378)	3.045^***^ (2.547–3.640)	3.302 ^***^ (2.675–4.075)	2.404 ^***^ (1.973–2.931)	2.704^***^ (2.178–3.356)	4.075^***^ (3.185–5.214)	5.582^***^ (4.332–7.192)

Variables in the model were school type, accommodation status, academic performance, pocket money, BMI (actual weight), weight perception and weight teasing

^*^P < 0.05; ^**^P < 0.01; ^***^P < 0.001

### Associations between weight perception, weight teasing and mental health among adolescents of different BMIs

As indicated in Table 5, for adolescents with low actual weight, the misperception of being overweight increased the risk of depressive symptoms (OR = 1.966, 95% CI: 1.034–3.736, *P* = 0.039) and moderate to severe anxiety symptoms (OR = 2.178, 95% CI: 1.023–4.638,* P* = 0.044). Among adolescents who were normal weight, incorrectly perceiving themselves as underweight or overweight increased the risk of mental health problems. Adolescents who perceived themselves as underweight but were actually overweight/obese were more likely to suffer from depressive symptoms (OR = 2.108, 95% CI: 1.423–3.122, *P* < 0.001), loneliness (OR = 2.073, 95% CI: 1.464–2.936, *P* < 0.001), and moderate to severe anxiety symptoms (OR = 2.709, 95% CI: 1.699–4.321, *P* < 0.001). However, the perception of about the right weight had no effect on and even served as a protection for experiencing loneliness among adolescents who were actually underweight (OR = 0.669, 95% CI: 0.471–0.952, *P* = 0.026) or overweight/obese. Regardless of the actual weight, adolescents who experienced weight teasing at least once a year were more likely to develop all three mental health problems. The experience of weight teasing more than once a year has the greatest impact on depressive symptoms (OR = 2.970, 95% CI: 2.325–3.793, *P* < 0.001), loneliness (OR = 3.839, 95% CI: 3.119–4.727, *P* < 0.001), mild anxiety symptoms (OR = 2.822, 95% CI: 2.236–3.562, *P* < 0.001) and moderate to severe anxiety symptoms (OR = 5.212, 95% CI: 3.846–7.065, *P* < 0.001) among overweight/obese adolescents.

**Table 5:** Associations between weight perception, weight teasing and mental health among adolescents of different BMIs

		Depressive symptoms		Loneliness		Mild Anxiety symptoms		Moderate to Severe Anxiety symptoms	
		OR (95% CI)	*P*	OR (95% CI)	*P*	OR (95% CI)	*P*	OR (95% CI)	*P*
**BMI underweight**									
Weight perception	Underweight	Ref		Ref		Ref		Ref	
	About the right weight	0.666 (0.404–1.097)	0.110	0.669 (0.471–0.952)	0.026	0.681 (0.439–1.057)	0.087	0.904 (0.518–1.578)	0.724
	Overweight	1.966 (1.034–3.736)	0.039	0.964 (0.521–1.786)	0.908	1.103 (0.534–2.280)	0.790	2.178 (1.023–4.638)	0.044
Weight teasing	Never	Ref		Ref		Ref		Ref	
	Less than once a year	0.995 (0.493–2.005)	0.988	3.272 (1.829–5.855)	< 0.001	3.149 (1.766–5.614)	< 0.001	2.105 (0.920–4.816)	0.078
	More than once a year	1.919 (1.105–3.333)	0.021	2.361 (1.417–3.935)	0.001	2.567 (1.483–4.444)	0.001	2.881 (1.442–5.757)	0.003
**BMI normal**									
Weight perception	About the right weight	Ref		Ref		Ref		Ref	
	Underweight	1.525 (1.251–1.859)	< 0.001	1.458 (1.256–1.692)	< 0.001	1.331 (1.118–1.585)	0.001	1.725 (1.359–2.190)	< 0.001
	Overweight	1.319 (1.114–1.562)	0.001	1.392 (1.222–1.585)	< 0.001	1.379 (1.192–1.596)	< 0.001	1.404 (1.144–1.723)	0.001
Weight teasing	Never	ref		ref		ref		ref	
	Less than once a year	1.734 (1.413–2.128)	< 0.001	2.306 (1.925–2.763)	< 0.001	1.818 (1.509–2.191)	< 0.001	2.074 (1.610–2.672)	< 0.001
	More than once a year	2.716 (2.230–3.308)	< 0.001	2.812 (2.313–3.420)	< 0.001	2.285 (1.862–2.805)	< 0.001	4.723 (3.729–5.983)	< 0.001
**BMI overweight/obese**									
Weight perception	Overweight	Ref		Ref		Ref		Ref	
	About the right weight	0.819 (0.582–1.151)	0.250	0.975 (0.767–1.239)	0.835	0.996 (0.748–1.327)	0.981	0.670 (0.425–1.057)	0.085
	Underweight	2.108 (1.423–3.122)	< 0.001	2.073 (1.464–2.936)	< 0.001	1.425 (0.941–2.157)	0.094	2.709 (1.699–4.321)	< 0.001
Weight teasing	Never	ref		ref		ref		ref	
	Less than once a year	1.042 (0.765–1.419)	0.796	2.388 (1.911–2.984)	< 0.001	1.718 (1.337–2.208)	< 0.001	1.474 (1.009–2.152)	0.045
	More than once a year	2.970 (2.325–3.793)	< 0.001	3.839 (3.119–4.727)	< 0.001	2.822 (2.236–3.562)	< 0.001	5.212 (3.846–7.065)	< 0.001

Variables in the model were sex, school type, accommodation status, academic performance, pocket money, weight perception and weight teasing

## Discussion

This study first reported the association between loneliness and weight perception as well as weight teasing in a large-scale Chinese adolescent population. In addition, the study also reported the latest prevalence of depressive symptoms, anxiety symptoms and loneliness in Chinese adolescents, and further explored their associations with weight perception and weight teasing after stratified by sex and weight, which identified both weight perception and weight teasing are independent risk factors for depressive symptoms, anxiety symptoms and loneliness.

This study found that the prevalence of depressive symptoms and anxiety symptoms among adolescents in Shanghai, China, was 18.0% and 38.8% respectively, with the number of girls outweighing that of boys. Compared to another Chinese study, the prevalence of depressive symptoms was low and the prevalence of anxiety symptoms was high [2]. Worldwide, there are a lot of children and adolescents who suffer from mental disorders [32]. According to a recent study, the overall prevalence of mental disorders among Chinese children and adolescents was 17.5%, and as age goes up from younger to 12–13 years, the overall prevalence of mental disorders shifted from a high prevalence among boys to a high prevalence among girls. This is likely multifactorial to include individual, environmental, and biological factors which cause girls to have more mental health problems when growing up, which is consistent with our findings [33]. Furthermore, the prevalence of loneliness among adolescents was 53.8%, higher than that of depression and anxiety, requiring the attention of society. In recent years, the prevalence of loneliness among adolescents has gradually increased worldwide [34], possibly due to the popularity of smartphones and the Internet. To be specific, adolescents' social behavior was changing and online communication was increasing [34]. It has been reported that a higher level of loneliness among adolescents was related to worsening mental health difficulties [35], and loneliness was also a predictor of future depression and anxiety in children and adolescents [36].Adolescent mental health problems are on the rise, and more research is needed to provide support for early prevention and intervention.

Another major finding of the study is that weight perception influenced adolescent mental health, independent of the actual weight, which is similar to the findings of a recent cross-sectional study [14]. Notably, after adjusting for weight perception and weight teasing, adolescents' overweight/obese weight status was a protective factor for mental health problems. This could be explained by the "fat and jolly hypothesis", which refers to that once adolescents become overweight or obese, they give up the intense struggle against excess weight and have an adaptive shift in their perception of physical appearance, preventing the development of poor mental health [37]. In this study, both boys and girls who perceived them as underweight or overweight were found to have higher risks of mental health problems. It was also discovered in a survey of 64,229 15-year-old adolescents from 47 nations that perceiving oneself as too thin or too fat was associated with poorer mental health, regardless of actual weight [38]. Adolescents who overestimated their weight were more likely to experience depression, anxiety, and stress [16, 39], while having underweight perception was associated with less psychological distress [40, 41], which was contrary to our result. A published study focused on body weight and loneliness in British adolescents reported that higher body weight was associated with increased loneliness [42]. However, few studies concern weight perception and loneliness. In this study, after controlling actual body weight, we found that underweight and overweight perception were independently associated with a higher risk of loneliness. Previous study has indicated that adolescents with non-normal weight perception had fewer confidants, which might increase the risk of loneliness, thus further studies were in need to explore it [43]. In the majority of studies, underweight perception was associated with worse psychological outcomes only among boys [16, 44]. This might be due to that boys prefer to be physically stronger and more muscular [44]. However, other studies have also indicated that perceiving oneself as underweight is associated with a higher rate of clinical psychological symptoms among both boys and girls [14, 45, 46], which is similar to our study. This could be attributed to the physical discrepancy between the self and more physically developed peers in late adolescence, which made adolescents who perceived themselves to be underweight psychologically worse [47]. Furthermore, a possible explanation for this might be that the growing acceptance of overweight and obesity has led to a shift in attitudes toward normal body shape [48, 49]. Meanwhile, only girls who perceived themselves as overweight were more likely to have depressive symptoms, which is consistent with the findings of previous research [50, 51]. This could be due to societal pressure regarding the current ideal body shape that girls intended to be slim and were more likely to experience body dissatisfaction than boys [52].

The current study also discovered that weight misperception increased the risk of mental health problems in adolescents. This finding is corroborated by a longitudinal study from the UK, demonstrating that adolescents with normal weight who misperceived themselves as underweight or overweight were more likely to experience clinical psychological symptoms than adolescents who accurately perceived their weights [45]. Despite the fact that a number of studies have shown the relationship between adolescents' misperception of their weight and mental health problems [20, 53, 54], this study found that among underweight and overweight/obese adolescents, inaccurately perceiving themselves as about the right weight was not negatively associated with their mental health or even produced a marginally protective effect. This might be explained by a study on American adolescents which suggested that accurate weight perception of being overweight or obese might increase awareness and sensitivity of weight stigma, while inaccurately having perceived themselves as about the right weight might act as a protective factor against depression in overweight and obese adolescents [41]. Similarly, adolescents who perceived themselves to be of normal weight but were actually underweight appeared to have better mental health [55]. This shows the possible protective effect of perceived normal weight on adolescents' mental well-being. In addition, a study discovered that among Asian youth, extreme weight misperception can be detrimental to mental health [41]. That is to say, overweight/obese individuals perceiving themselves to be underweight had a higher risk for depression [41], which is consistent with our findings.

One more finding is that weight teasing had a greater impact on depressive symptoms, loneliness, and anxiety symptoms, among adolescents compared to body weight status and weight perception. Moreover, it was found that the more frequently adolescents experienced weight teasing, the greater the harmful effect was. It has been proposed that eating disorders could mediate the relationship between weight stigma and psychological problems (depression and anxiety) [56]. Experiencing weight stigma was observed to be associated with higher risk of loneliness in adults [57]. Moreover, a cohort study reported that weight discrimination, but not actual weight of adolescents of six grade increased the risk of loneliness in two years later [58]. Our results showed that weight teasing was independently associated with loneliness in Chinese adolescents after adjusting for actual weight and weight perception, which consists with previous study. The reason why weight teasing was more likely to cause mental health problems in adolescents may be that exposure to information related to weight stigma will increase weight concerns and lead to poor mental health, regardless of actual weight and weight perception [59, 60]. Furthermore, the experience of weight teasing changes adolescents' perception of weight or increased internalization of weight stigma, and the effect of weight perception and weight teasing is superimposed, thus resulting in an unhealthy mental state [14, 59]. Our study also found that girls who experienced weight teasing less than once a year were at elevated risk of depressive symptoms, whereas no significant results were observed in boys. Girls may be more sensitive to teasing feedback from male peers about their weight, leading to lower self-esteem about their appearance [61], and low self-esteem is a vulnerability marker of depression [62]. In addition, this study discovered that frequent weight teasing had the greatest impact on the mental health of overweight and obese adolescents, compared to those adolescents with normal weight. First, weight teasing was more common among overweight and obese adolescents [63]. Second, social media has been influencing social beliefs and attitudes. Research has shown that the media could exacerbate weight stigma by reinforcing ideal body image, which could lead to deterioration in mental health [64]. In addition, it has been found that in overweight or obese individuals, frequent experience of weight stigma increased the internalization of weight bias and thus made them more likely to adopt inappropriate coping responses (e.g. negative self-talk, withdrawal, and avoidance behaviors) to weight stigma, worsening their psychological state and increasing their risk of experiencing depression, anxiety, and stress [65]. Thus, adolescent mental health may be improved by reducing weight teasing and adopting more adaptive coping responses.

## Limitation

This study does have several limitations. First, this study is a cross-sectional study that cannot verify causality. Longitudinal studies are needed to further explore the causative relationship. Second, all the data collected in the study were self-reported, which may be subject to respondent bias. Future studies may consider using objective measurement methods to enhance the reliability of the survey data. Finally, the participants of the study were adolescents in Shanghai, which cannot represent China as a whole, so the sample representation is limited.

## Conclusion

This study found that the prevalence of mental health problems among adolescents was high in Shanghai, China, especially loneliness. Weight perception and weight teasing, not actual weight, independently affected the mental health of Chinese adolescents, with weight teasing having a greater negative impact. Weight misperception also increased the risk of poor mental health. It calls for greater emphasis on promoting a healthy weight perception to improve mental health among adolescents.

## Data Availability

The datasets used during the current study are available from the corresponding author on reasonable request.

## Abbreviations

BMI: Body Mass Index
ULS-6: The UCLA Loneliness Scale Short Version
CNY: Chinese Yuan
OR: Odds Ratio
CI: Confidence Interval
GAD-7: The Generalized Anxiety Disorder-7
PHQ-2-C: The Chinese Version of the Patient Health Questionnaire-2
